# Unusual Native Valve Remnant in the Left Ventricular Outflow Tract After Valve-in-Ring Transcatheter Mitral Valve Replacement

**DOI:** 10.3390/jcm15051732

**Published:** 2026-02-25

**Authors:** Sergio Enea Masnaghetti, Fabiana Isabella Gambarin, Stefano Maffè, Marco Gnemmi, Michela Conti, Andrea Audo, Massimo Pistono

**Affiliations:** 1 Laboratory for the Study of Exercise and Cardiorespiratory Signals, Cardiac Rehabilitation Unit, Istituti Clinici Scientifici Maugeri IRCCS, Veruno Institute, 28013 Gattico-Veruno, Italy; sergio.masnaghetti@icsmaugeri.it (S.E.M.); marco.gnemmi@icsmaugeri.it (M.G.); michela.conti@icsmaugeri.it (M.C.); massimo.pistono@icsmaugeri.it (M.P.); 2 Division of Cardiology, SS. Trinità Borgomanero Hospital, Azienda Sanitaria Locale Novara, 28021 Borgomanero, Italy; stemaffe@libero.it; 3 Cardiac Surgery Department, Azienda Ospedaliero-Universitaria Santi Antonio e Biagio e Cesare Arrigo, 15121 Alessandria, Italy; aaudo@ospedale.al.it

**Keywords:** valve-in-ring, transcatheter heart valve replacement, native mitral valve remnant, 3D transesophageal echocardiography, structural heart disease

## Abstract

**Background and clinical significance**. Valve-in-ring (ViR) transcatheter mitral valve replacement (TMVR) is an established therapeutic option for patients with failed surgical mitral valve repair at high surgical risk. Left ventricular outflow tract (LVOT) obstruction and prosthesis-related complications are well described, but other postprocedural findings remain poorly characterized. **Case presentation**. We report a challenging case of a persistent LVOT mass following ViR TMVR. A 78-year-old man underwent transapical ViR TMVR with a Sapien 3 valve for mitral stenosis. Early post-procedural echocardiography showed normal prosthetic function and no LVOT obstruction. During inpatient cardiac rehabilitation, transthoracic echocardiography revealed a mobile mass in the LVOT. The patient did not show any clinical, microbiological, or laboratory evidence of infection. Blood cultures were negative, and the mass showed no changes despite anticoagulation. Two- and three-dimensional transesophageal echocardiography demonstrated a mobile structure attached to the mitral prosthetic ring by a thin peduncle. After a comprehensive multimodality assessment, thrombus and infective endocarditis were considered unlikely. The mass was ultimately interpreted as a displaced remnant of the native anterior mitral leaflet. Given the prohibitive surgical risk and absence of complications, conservative management with echocardiographic follow-up was adopted. **Conclusions**. This case study emphasizes the role of advanced echocardiography and multimodality analysis in avoiding misdiagnosis and inappropriate therapeutic interventions.

## 1. Introduction

Valve-in-ring (ViR) transcatheter mitral valve replacement (TMVR) has emerged as a valuable therapeutic option for patients with failed surgical mitral valve repair who are deemed at high or prohibitive surgical risk. Data from prospective trials and large registries have demonstrated acceptable short- and mid-term clinical and hemodynamic outcomes in carefully selected patients [1,2]. Nevertheless, ViR TMVR remains a complex procedure, and both acute and late complications continue to be reported.

Among the most detrimental complications, left ventricular outflow tract (LVOT) obstruction has been extensively described, particularly in patients with a long or redundant anterior mitral leaflet. The interaction between preserved native leaflet tissue and the implanted transcatheter valve may lead to dynamic LVOT compromise, prompting the development of preventive techniques such as intentional anterior leaflet modification [3,4]. Other reported complications include valve malposition, residual mitral regurgitation, paravalvular leak, and prosthesis dysfunction [5].

However, the presence of a persistent mobile mass within the LVOT after ViR TMVR, mimicking thrombus or infective endocarditis and ultimately suspected to represent a remnant of the native mitral valve, has not been previously described as a distinct post-procedural mass-like finding. We report a diagnostically challenging case that highlights the importance of multimodality imaging and careful differential diagnosis following ViR TMVR.

## 2. Case Presentation

A 78-year-old man with a history of surgical mitral valve repair performed in 2001 was referred for worsening exertional dyspnea due to progressive mitral valve stenosis. The patient had permanent atrial fibrillation and was receiving chronic oral anticoagulation with warfarin. Given the high operative risk due to coexisting comorbidities (including chronic obstructive pulmonary disease and chronic hemolytic anemia), the Heart Team recommended a transcatheter approach. On 19 May 2025, the patient underwent ViR TMVR via a transapical approach using a balloon-expandable Edwards Sapien 3 bioprosthesis (29 mm) (Edwards Lifesciences Pty Ltd., North Ryde, Australia). The procedure was uneventful, with optimal valve positioning and no immediate peri-procedural complications.

A few days before TMVR, the patient had undergone percutaneous coronary intervention with drug-eluting stent implantation in the left anterior descending artery and first diagonal branch during hospitalization for acute heart failure. As such, the patient was under double antiplatelet therapy in addition to postoperative oral anticoagulation.

Early postoperative transthoracic echocardiography (TTE) demonstrated normal prosthetic valve function, no residual mitral regurgitation, and no evidence of LVOT obstruction. Severe tricuspid regurgitation was noted but was not associated with hemodynamic instability.

On 30 May 2025, the patient was admitted to an in-hospital cardiac rehabilitation program. At admission, he was markedly deconditioned, predominantly bedridden, and dependent in all activities of daily living. Routine entry TTE revealed an iso-echogenic, mobile, pedunculated mass located near the aortic valve plane and protruding into the LVOT. The precise site of attachment could not be clearly identified. The mass measured approximately 11 × 9 × 17 mm.

Throughout hospitalization, the patient remained asymptomatic. No fever or signs of systemic infection were observed. Laboratory investigations showed normal procalcitonin levels and only mild, non-specific elevation of inflammatory markers. Blood cultures were repeatedly negative, except for a single isolate of *Staphylococcus epidermidis*, which was considered a sign of contamination.

Given the possibility of a thrombotic origin, the adequacy of oral anticoagulation with warfarin was carefully reassessed; however, as the patient was already within the therapeutic INR range, no changes in antithrombotic therapy were made. A follow-up TTE performed after approximately two weeks of anticoagulation (with INR in therapeutic range) showed no change in the size or morphology of the mass.

**Figure 1:**
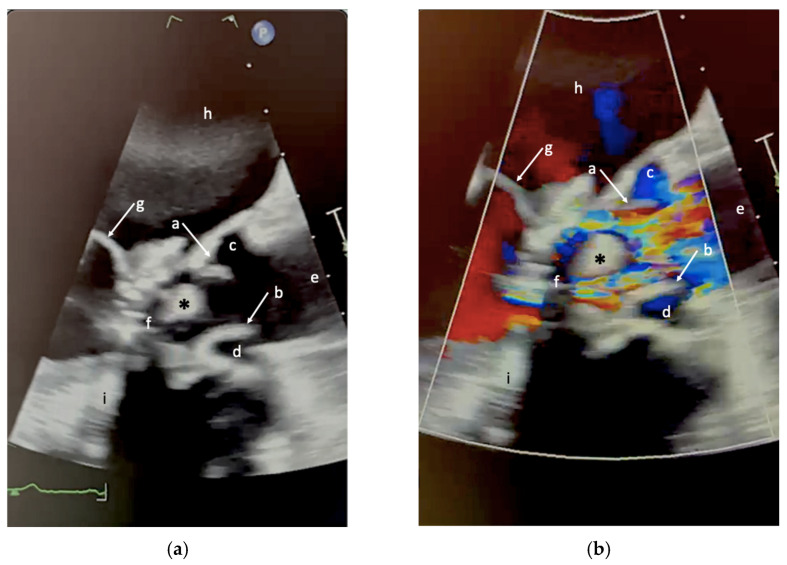
Transesophageal echocardiography of the left ventricular outflow tract (LVOT) acquired during systole, illustrating the anatomical relationships between a mobile intracardiac mass (*) and adjacent cardiac structures. (**a**) Two-dimensional (2D) B-mode image. (**b**) 2D color Doppler image. Both panels show: a = right coronary aortic cusp; b = non-coronary aortic cusp; c = right sinus of Valsalva; d = non-coronary sinus of Valsalva; e = ascending aorta; f = LVOT; g = mitral leaflet; h = left atrium; i = acoustic shadowing from the prosthetic mitral annulus. The green line at the bottom represents a portion of the single-ECG lead recorded during the echocardiographic examination.

**Figure 2:**
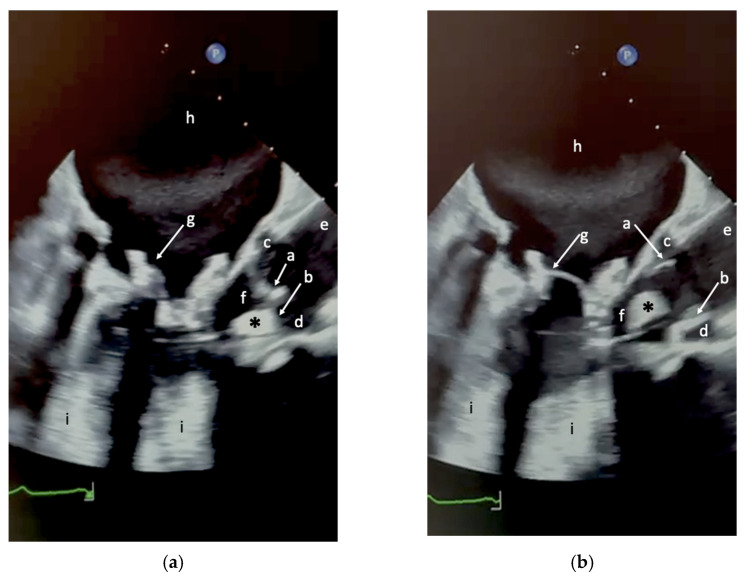
Transesophageal echocardiography of the left ventricular outflow tract (LVOT) illustrating the anatomical relationships between the mobile intracardiac mass (*) and adjacent cardiac structures during diastole and systole. (**a**) Two-dimensional (2D) B-mode image in diastole. (**b**) 2D B-mode image in systole. Anatomical labels (a–i) correspond to those reported in Figure 1. The green line at the bottom represents a portion of the single-ECG lead recorded during the echocardiographic examination.

A transesophageal echocardiogram performed one more week later (TEE, Figure 1 and Figure 2) confirmed the presence of a mildly hyperechogenic mobile mass.

**Figure 3:**
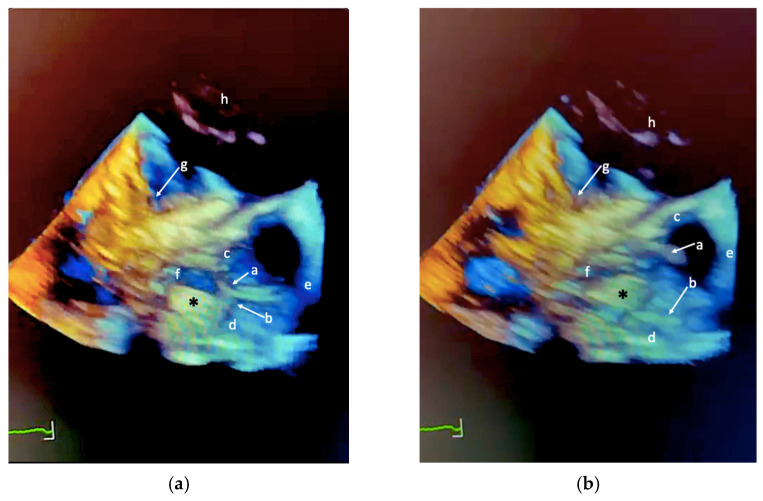
3D transesophageal echocardiography of the left ventricular outflow tract (LVOT) illustrating the anatomical relationships between the mobile intracardiac mass (*) and adjacent cardiac structures during diastole and systole. (**a**) Two-dimensional (2D) B-mode image in diastole. (**b**) 2D B-mode image in systole. Anatomical labels (a–h) correspond to those reported in Figure 1 and Figure 2. The green line at the bottom represents a portion of the single-ECG lead recorded during the echocardiographic examination.

However, acoustic shadowing from the mitral prosthetic ring limited accurate identification of its anchoring site. To improve anatomical characterization, a three-dimensional TEE (Figure 3 and Supplementary Material Video S1) was subsequently performed using a different imaging platform. This examination revealed a highly mobile mass attached to the mitral prosthetic ring by a very thin peduncle, extending toward the LVOT. Although the initial appearance raised concern for infective endocarditis, further multiparametric evaluation favored an alternative diagnosis.

A final TTE before discharge confirmed persistence and stability of the mass, with no evidence of embolization or hemodynamic compromise. Given the patient’s prohibitive surgical risk, reintervention was not recommended by the Heart Team. The patient was discharged at home with triple anticoagulation therapy (double antiplatelet therapy with acetylsalicylic acid and clopidogrel, and anticoagulation with warfarin). According to current postoperative recommendations for mitral valve surgery [6] and given the concomitant indication for anticoagulation due to atrial fibrillation [7], no additional anticoagulation was prescribed specifically to prevent thrombus formation on the suspected mitral valve remnant.

At follow-up, a telephone interview revealed that the patient died on 28 August 2025 due to a pulmonary infection and its related complications in the context of multiple comorbidities and advanced frailty, and not as a consequence of the underlying cardiac condition. A postmortem examination was not performed because of explicit refusal by the patient’s family.

## 3. Discussion

This case illustrates a rare and diagnostically challenging finding following ViR TMVR: a persistent mobile mass protruding into the LVOT. The main differential diagnoses included thrombus formation, infective endocarditis, native mitral valve remnants, and, less likely, a primary cardiac tumor.

A thrombotic origin was initially considered because of its potential reversibility. However, several findings argued against this hypothesis. The LVOT is characterized by high-velocity blood flow, making thrombus persistence unlikely. Moreover, the mass remained unchanged after prolonged therapeutic anticoagulation, and its echocardiographic appearance was not typical of thrombotic material.

Infective endocarditis was also considered, particularly given the highly mobile and pedunculated morphology observed on three-dimensional TEE. Nevertheless, the complete absence of clinical signs of infection, persistently negative blood cultures, normal procalcitonin levels, and only mild, non-specific inflammatory marker elevation strongly argued against this diagnosis.

The hypothesis of a remnant of the native mitral valve (most likely a displaced portion of the anterior leaflet) appeared the most plausible explanation. Preservation of native leaflet tissue during TMVR is a well-recognized contributor to LVOT-related complications, and strategies aimed at reducing leaflet interference have been developed [3,4]. While the literature has largely focused on dynamic LVOT obstruction, little attention has been paid to the potential for leaflet remnants to assume abnormal positions after ViR TMVR.

In this context, even accurate pre-procedural planning and comprehensive imaging assessment of leaflet anatomy could not reliably predict the final spatial configuration of the native leaflet after valve deployment. Although an excessively long anterior mitral leaflet may theoretically predispose to displacement toward the LVOT, this event remains difficult to anticipate with certainty and, importantly, cannot be completely prevented. Pre-implant imaging allows accurate characterization of baseline anatomy but does not fully capture the complex leaflet–prosthesis interactions that occur during and after device expansion.

In this patient, the transapical approach was selected, which provides a more direct trajectory to the mitral annulus compared with the transseptal route, where valve advancement and positioning are technically more demanding. This approach facilitates limited leaflet manipulation during deployment, a key factor in minimizing the risk of anterior leaflet injury and subsequent post-procedural displacement.

In our case, the echocardiographic texture of the mass, its stability over time, resistance to high-flow conditions, and lack of response to anticoagulation support a fibrous or calcified structure rather than thrombus or vegetation. Late complications related to preserved native mitral tissue have previously been described in surgical series, lending further plausibility to this interpretation [8].

Advanced imaging modalities, such as cardiac magnetic resonance or positron emission tomography, were considered. However, given the patient’s advanced age, frailty, and the limited impact of additional imaging on management, a conservative strategy based on serial echocardiographic follow-up was adopted. Despite the absence of embolic events, the persistence of a mobile LVOT mass implies a potential embolic risk, warranting close surveillance.

To our knowledge, this represents the first detailed description of a suspected native mitral valve remnant presenting as a mobile LVOT mass after valve-in-ring TMVR, a finding that appears to be rare and poorly characterized.

The main limitation of this report is the absence of a postmortem examination, which could have definitively clarified the nature of the mass. Nevertheless, infective endocarditis appears highly unlikely, as the patient did not receive antimicrobial therapy after discharge and remained clinically free from signs of infection for almost three months. An autopsy would have allowed definitive differentiation between a native mitral valve remnant and thrombotic material. In the absence of histopathological confirmation, the final diagnosis remains presumptive.

## 4. Conclusions

This case underscores the complexity of post-procedural findings after ViR TMVR and the importance of careful differential diagnosis when a mobile intracardiac mass is detected. Multimodal echocardiography, particularly three-dimensional TEE, is crucial for distinguishing among thrombus, vegetation, and native tissue remnants. Awareness of this potential complication may help avoid unnecessary or inappropriate therapeutic interventions.

## Data Availability

The data presented in this study are available on request from the corresponding author to ensure the privacy of the patient included.

## Supplementary Materials

The following supporting information can be downloaded at https://www.mdpi.com/article/10.3390/jcm15051732/s1. Video S1: 3D transesophageal echocardiography with mobile intracardiac mass.

## Abbreviations

The following abbreviations are used in this manuscript:

2D	two-dimensional
3D	three-dimensional
LVOT	left ventricular outflow tract
TEE	transesophageal echocardiogram
TMVR	transcatheter mitral valve replacement
TTE	transthoracic echocardiography
ViR	valve-in-ring
